# *SF3B1* mutated MDS: Blast count, genetic co-abnormalities and their impact on classification and prognosis

**DOI:** 10.1038/s41375-022-01728-5

**Published:** 2022-10-19

**Authors:** Sandra Huber, Torsten Haferlach, Manja Meggendorfer, Stephan Hutter, Gregor Hoermann, Constance Baer, Wolfgang Kern, Claudia Haferlach

**Affiliations:** MLL Munich Leukemia Laboratory, Max-Lebsche-Platz 31, 81377 Munich, Germany

**Keywords:** Myelodysplastic syndrome, Cancer genomics

## Abstract

Recently, MDS with mutated *SF3B1* and blast count <5% was proposed as distinct entity with favorable prognosis by the international working group for the prognosis of MDS (IWG-PM), the 5th edition of the WHO classification and the International Consensus Classification. To further characterize this entity with respect to the genomic landscape, AML transformation rate and clinical outcome, we analyzed 734 MDS patients by whole genome sequencing. *SF3B1* mutations were identified in 31% (*n* = 231), most frequently accompanied by *TET2* mutations (29%). 144/231 (62%) *SF3B1*^mut^ samples fulfilled entity criteria proposed by IWG-PM (*SF3B1*ent). These cases were associated with longer survival, lower AML transformation rate, normal karyotypes and harbored less accompanying mutations compared to *SF3B1*^mut^ samples not falling into the proposed *SF3B1* entity (*SF3B1*nent). Of *SF3B1*^mut^ cases 7% (15/231; *SF3B1*ent: 3/144 [2%]; *SF3B1*nent: 12/87 [14%]) progressed to AML compared to 15% *SF3B1* wild-type patients (75/503). Of these 15 *SF3B1*^mut^ cases, 10 (67%) showed *RUNX1* mutations at MDS or AML stage. Multivariate analysis revealed that del(5q) and *RUNX1* mutations were independent negative prognostic factors for overall survival, while blast count >5% was not. In conclusion, *SF3B1*^mut^ MDS has a favorable prognosis independent of blast count if karyotype and *RUNX1* mutations are considered.

## Introduction

Myelodysplastic neoplasms (MDS) are clonal disorders characterized by peripheral cytopenias, morphologic dysplasia in hematopoietic cells and ineffective hematopoiesis [1, 2]. The currently used revised 4th edition of the WHO classification (WHO 2017) in MDS is mainly based on the number of cytopenias, dysplastic lineages, and the percentage of ring sideroblasts (RS) and blasts detected in bone marrow and peripheral blood samples [3]. Within the last years, the use of next generation sequencing (NGS) enabled the identification of driver genes in MDS providing insights into the underlying heterogeneous genetic landscape [4–6]. In this line, about half of MDS patients harbor somatic mutations in splicing pathway genes. Of these, *SF3B1* is the most commonly mutated gene and if mutated shown to be associated with RS, higher white blood cell counts and lower bone marrow blasts [6–9]. Moreover, *SF3B1* mutations define a distinct MDS subset showing favorable prognosis and indolent disease course [10]. Thus, in the WHO 2017 the *SF3B1* mutation is integrated into the diagnosis of MDS-RS (diagnostic criteria: RS ≥ 15% or RS ≥ 5% if *SF3B1*^mut^) [1].

Following up on this, the international working group for the prognosis of MDS (IWG-PM) proposed MDS with mutated *SF3B1* as a distinct entity if certain criteria are fulfilled (Supplementary Table S1) [11]. These criteria included: (1) cytopenia as defined by standard hematologic values, (2) somatic *SF3B1* mutation, (3) morphologic dysplasia (with or without RS), (4) bone marrow blasts <5% and peripheral blood blasts <1%, and (5) WHO 2017 criteria for MDS 5q-, MDS/MPN-RS-T, or other MDS/MPN or MPN are not met. Further exclusion criteria were: (1) poor-risk cytogenetics comprising monosomy 7, inv(3) or abnormalities of chromosome 3q26, and complex karyotype (≥3 chromosomal abnormalities); and (2) accompanying mutations in *RUNX1* and/or *EZH2*. The presence of *JAK2*V617F, *CALR*, or *MPL* mutations would strongly support the diagnosis of MDS/MPN-RS-T.

The upcoming 5th edition of the WHO Classification (WHO 2022) emphasizes a genetic basis for defining diseases and has now categorized MDS into morphologically defined MDS and MDS with defining genetic abnormalities (DGA) while largely abandoning the blast cut-off between MDS and AML if AML DGA are present [2]. It has further incorporated many of the proposed IWG-PM criteria into the newly introduced entity “MDS with low blasts and *SF3B1* mutation” [2]. However, according to WHO 2022 only biallelic *TP53* inactivations are excluded besides certain cytogenetic abnormalities (Supplementary Table S1). In contrast to the WHO 2022, the International Consensus Classification (ICC) requires an *SF3B1* variant allelic frequency (VAF) ≥ 10% in the absence of certain cytogenetic abnormalities, *RUNX1* and multi-hit *TP53* (Supplementary Table S1) [12]. It further sets the blast cut-off for AML-DGA to 10%, while cases with 10–19% blasts without DGA are assigned as a new category MDS/AML. In this study, we defined the *SF3B1* entity (*SF3B1*ent) based on the first publication proposed by the IWG-PM, but also discuss the changes in classification according to 5th edition of the WHO classification and ICC.

The aim of the study was to analyze the *SF3B1* mutation and the proposed *SF3B1* entity in a large cohort of 734 MDS patients with respect to the incidence, genomic landscape, AML transformation rate and clinical outcome.

## Material and methods

### Patients cohort and samples

For this analysis, we selected 734 MDS samples with material available to perform whole genome sequencing sent to the MLL Munich Leukemia Laboratory between 09/2005 and 12/2019. Diagnoses (from peripheral blood and bone marrow) were made based on cytomorphology, cytogenetics and molecular genetics as previously published [13–15]. All cases were classified into specific subgroups according to WHO 2017 [16]. For abbreviations of entities, see Table 1. Therapy-related MDS were excluded from this study. The MDS cohort comprised 310 (42%) female and 424 (58%) male cases with a median age of 73 years (range: 23–93 years) and a median follow-up of 9.3 years. All patients gave their written informed consent for genetic analyses and to the use of laboratory results as well as clinical data for research purposes according to the Declaration of Helsinki. The study was further approved by the laboratory´s institutional review board.

**Table 1:** WHO entities and *SF3B1* classification of the MDS cohort.

WHO 2017 Diagnosis	Number of samples, *n* (%)	*SF3B1*wt, *n* (%)	*SF3B1*^mut^, *n* (%)	*SF3B1*ent, *n* (% of *SF3B1*^mut^)	*SF3B1*nent, *n* (% of *SF3B1*^mut^)
MDS with single lineage dysplasia (MDS-SLD)	22 (3)	21 (95)	1 (5)	1 (100)	0 (0)
MDS with multilineage dysplasia (MDS-MLD)	105 (14)	104 (99)	1 (1)	1 (100)	0 (0)
MDS with single lineage dysplasia with ring sideroblasts (MDS-RS-SLD)	51 (7)	8 (16)	43 (84)	37 (84)	6 (16)
MDS with multilineage dysplasia with ring sideroblasts (MDS-RS-MLD)	149 (20)	21 (14)	128 (86)	105 (82)	23 (18)
MDS with isolated del(5q) (MDS 5q-)	107 (15)	86 (80)	21 (20)	0 (0)	21 (100)
MDS with excess blasts (MDS-EB-1)	149 (20)	124 (83)	25 (17)	0 (0)	25 (100)
MDS with excess blasts (MDS-EB-2)	151 (21)	139 (92)	12 (8)	0 (0)	12 (100)
MDS total	734	503 (69)	231 (31)	144 (62)	87 (38)

*wt* wild-type, *mut* mutated, *SF3B1ent* proposed *SF3B1* entity, *SF3B1nent* *SF3B1* mutated cases not meeting *SF3B1* entity criteria.

### Whole genome sequencing (WGS) and variant filtering

WGS analysis was performed for all patients. For this, total genomic DNA was extracted from lysed cell pellet of bone marrow or peripheral blood using the MagNA Pure 96 with DNA and Viral Nucleic Acid Large Volume Kit and Cellular RNA Large Volume Kit (Roche, Basel, Switzerland). Library preparation and sequencing as well as calling and filtering of single nucleotide variants, structural variants and somatic copy number variations (CNVs) were performed as previously described [17, 18]. Copy neutral loss of heterozygosity (CN-LOH) was assessed using HadoopCNV.

### Mutational analysis

In this study, we evaluated mutations in 73 genes associated with myeloid neoplasms for all patients from WGS data only or from combined WGS and targeted NGS panels (see supplementary material). Out of all 734 cases, 605 samples were additionally analyzed by targeted sequencing within a recent study [6] and 87 cases were analyzed by targeted NGS during routine diagnostics [19]. WGS data confirmed all mutations detected by targeted NGS panels and was further consulted for completing the mutational analysis of the 73 genes. The presence of *FLT3*-ITD and *KMT2A*-PTD were retrieved from WGS data only.

### Statistical analysis

Statistical analyses were performed using SPSS version 19.0 (IBM Corporation, Armonk, NY). Analyses for overall survival (OS) and cumulative incidence (CI) of disease progression were performed according to Kaplan-Meier and compared using two-sided log rank tests. The OS was calculated as time from diagnosis to death or last follow-up. For the CI of disease progression death was considered as a competing event. Between different groups numerical variables were compared using the Mann–Whitney*-U*-Test, and dichotomous variables using chi-square test. Cox proportional hazards regression model was used to identify the impact of different variables on OS or AML transformation. All results were considered significant at *p* < 0.05.

## Results

### Incidence and prognostic impact of SF3B1 mutations

**Fig. 1:**
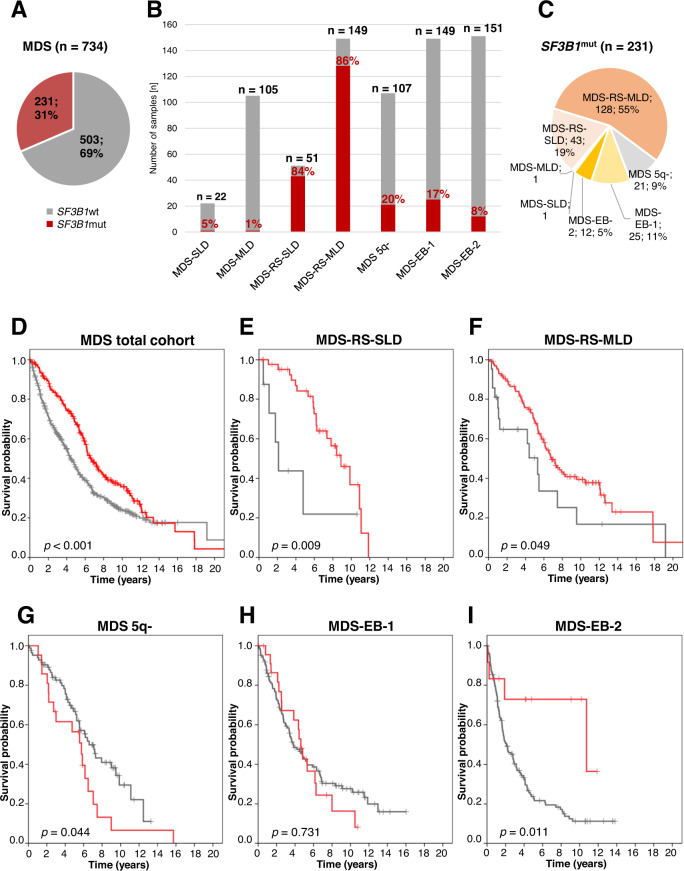
Distribution and OS of *SF3B1* mutations in MDS. **A** Frequency of *SF3B1* mutations in the entire MDS cohort; wt wild-type, mut mutated. **B** Proportion of *SF3B1*^mut^ cases within different MDS entities (red: mutated; gray: wild-type). **C** WHO 2017 entities of *SF3B1*^mut^ MDS. **D** OS of patients with mutated (*n* = 231; red) vs. wild-type (*n* = 503; gray) *SF3B1* within the entire MDS cohort. **E** OS of MDS-RS-SLD patients with mutated (*n* = 43; red) vs. wild-type (*n* = 8; gray) *SF3B1*. **F** OS of MDS-RS-MLD patients with mutated (*n* = 128; red) vs. wild-type (*n* = 21; gray) *SF3B1*. **G** OS of MDS 5q- patients with mutated (*n* = 21; red) vs. wild-type (*n* = 86; gray) *SF3B1*. **H** OS of MDS-EB-1 patients with mutated (*n* = 25; red) vs. wild-type (*n* = 124; gray) *SF3B1*. **I** OS of MDS-EB-2 patients with mutated (*n* = 12; red) vs. wild-type (*n* = 139; gray) *SF3B1*.

*SF3B1* mutations were identified in 231 of 734 (31%) MDS patients and were mainly found in MDS-RS (171/200; 86%; MDS-RS-SLD: 43/51, 84%; MDS-RS-MLD: 128/149; 86%) resulting in 74% (171/231) of all *SF3B1*^mut^ cases (Table 1; Fig. 1A–C). In addition, 13% (37/300) of MDS with excess blasts (MDS-EB-1/2) and 20% (21/107) of MDS 5q- harbored *SF3B1* mutations together accounting for 25% (58/231) of all *SF3B1*^mut^ cases (Table 1; Fig. 1B, C). The remaining 1% of *SF3B1*^mut^ cases were an MDS-SLD and an MDS-MLD sample. Of note, *SF3B1* mutations were most frequently found in patients with blast count <5% (192/419; 46%).

In the total MDS cohort *SF3B1* mutations were associated with better OS (median: 79 vs. 53 months; *p* < 0.001; Fig. 1D). Within the different MDS entities *SF3B1* mutations were favorable in MDS-RS-SLD (median OS: 106 vs. 25 months; *p* = 0.009), MDS-RS-MLD (median: 82 vs. 64 months; *p* = 0.049) and MDS-EB-2 (median: 129 vs. 25 months; *p* = 0.011), but were associated with a shorter OS in MDS 5q- (median: 69 vs. 79 months; *p* = 0.044) (Fig. 1E–I). Irrespective of the *SF3B1* mutation status, MDS-RS-SLD patients showed the best OS within the entire MDS cohort, while in contrast MDS with excess blasts was associated with the shortest OS (Supplementary Fig. S1A). A similar pattern was observed when focusing on *SF3B1* mutated (*SF3B1*^mut^) patients (Fig. S1B). However, the unusual long OS for *SF3B1* mutated MDS-EB-2 might be affected by therapy, in this regard allogeneic stem cell transplantation (SCT) received by 3/12 *SF3B1*^mut^ MDS-EB-2 patients (Supplementary Table S2).

**Fig. 2:**
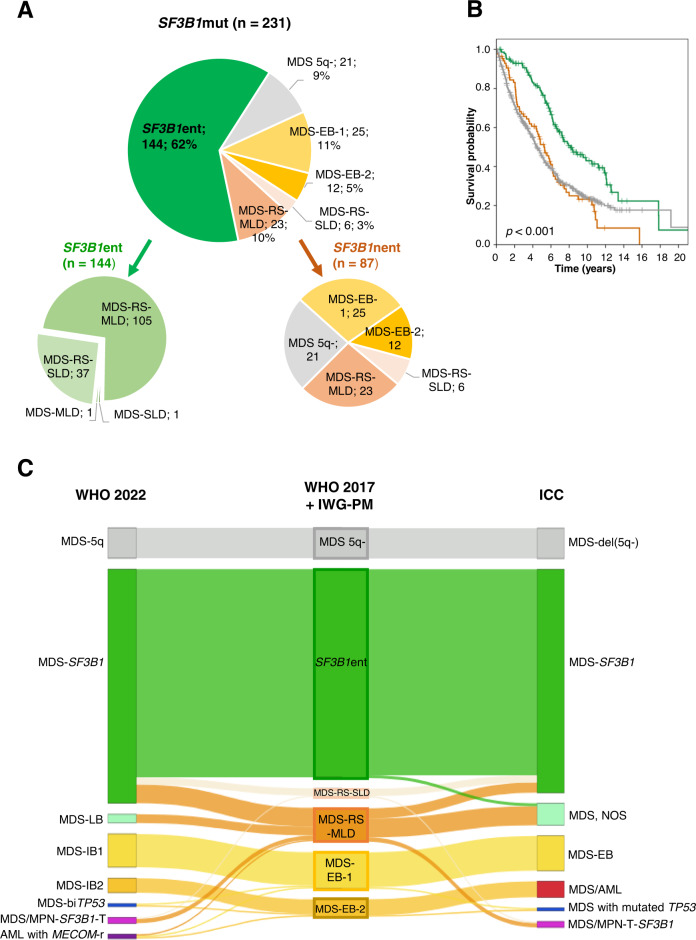
Categorization and OS of *SF3B1*^mut^ samples. **A** WHO 2017 entities of *SF3B1*^mut^ samples and classification into the IWG-PM proposed *SF3B1* entity (*SF3B1*ent) or non-*SF3B1* entity (*SF3B1*nent). **B** OS of patients with mutated *SF3B1* fulfilling criteria for proposed *SF3B1* entity (*n* = 144; green) or not (*n* = 87; brown) vs. wild-type *SF3B1* (*n* = 503; gray) (*p* < 0.001). **C** Comparison of *SF3B1*^mut^ MDS diagnoses based on the currently used revised 4th edition of the WHO (WHO 2017) and the IWG-PM criteria (middle) to the corresponding MDS diagnoses considering the upcoming 5th edition of WHO (WHO 2022; left) and the International Consensus Classification (ICC; right).

Within the *SF3B1*^mut^ cohort 144/231 (62%) samples fulfilled the criteria proposed by IWG-PM (*SF3B1*ent) (Table 1; Fig. 2A; Supplementary Fig. S2A). *SF3B1*ent cases had a longer OS compared to *SF3B1*^mut^ samples not falling into the proposed *SF3B1* entity (*SF3B1*nent) (Fig. 2B; median: 97 vs. 63 months; *p* < 0.001). However, no positive effect of *SF3B1* mutations on OS was observed within MDS-RS-SLD or MDS-RS-MLD if *SF3B1* non-entity mutated cases were compared to wild-type cases (Supplementary Fig. S3A, B). Of note, *SF3B1* mutations were associated with the presence of RS in both groups (*SF3B1*ent and *SF3B1*nent), showing median percentages of 63 and 49, respectively (Supplementary Fig. S2B; *p* < 0.001).

Differences in the defining criteria for the *SF3B1* entity between WHO 2022, ICC and IWG-PM lead to changes in assignment of 18 and 14 *SF3B1*^mut^ cases, respectively (Supplementary Table S1; Fig. 2C). A detailed analysis of the changes is described in the supplement.

### Recurrent SF3B1 mutations

**Fig. 3:**
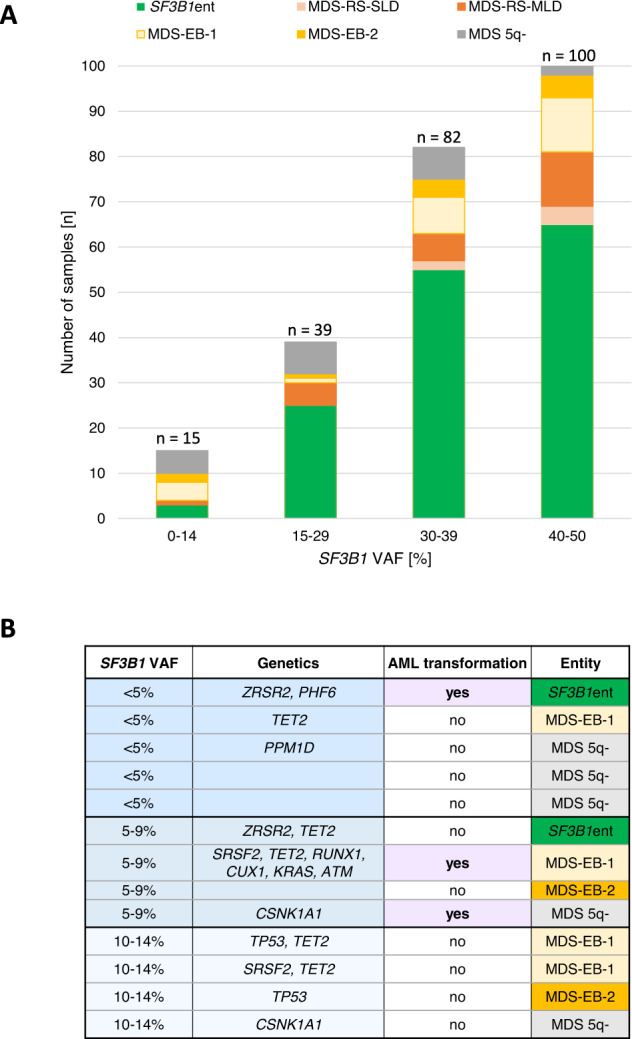
Variant allelic frequencies (VAFs) of 231 *SF3B1*^mut^ samples. **A** *SF3B1* VAFs with respect to the different entities; *n* (mutations) = 236. **B** Characteristics of cases having only one *SF3B1* mutation and a VAF below 15%.

Within the entire cohort, 25 different *SF3B1* mutations, most frequently affecting amino acid K700 (53%, 123/231), were detected with a mean VAF ranging from 22% to 48% (Supplementary Fig. S4). In 5/231 patients two different *SF3B1* mutations were detected resulting in 236 *SF3B1* mutations in total (Table in Supplementary Fig. S4B). The VAF of each *SF3B1* mutation did not exceed 50% (range: 4–50%) (Fig. 3A). Of all *SF3B1* mutations 77% (182/236) showed a VAF > 30% with *SF3B1*ent accounting for 66% (120/182). Moreover, 17% (39/236) showed a VAF between 15% and 29%, mainly belonging to *SF3B1*ent (25/39, 64%). *SF3B1* VAFs <15% were seen in 15 cases, rarely in *SF3B1*ent (20%, 3/15). However, two of those 15 cases (one *SF3B1*ent; one MDS-RS-MLD) showed a second *SF3B1* mutation with a VAF > 20% (Supplementary Fig. S4B). Of the remaining cases having *SF3B1* mutations with VAFs <15% (*n* = 13), 5/13 (39%) samples were MDS 5q-, 6 (46%) MDS-EB-1/2 and 2 (15%) were *SF3B1*ent (Fig. 3B). Of note, no CNVs or CN-LOHs overlapping with *SF3B1* were found.

### Genomic landscape of SF3B1^mut^ patients

Regarding cytogenetic abnormalities, 69/231 (30%) *SF3B1*^mut^ samples showed aberrant karyotypes (*SF3B1*ent: 27/144, 19%; *SF3B1*nent: 42/87, 48%; *p* < 0.001; Supplementary Fig. S5A). Notably, cytogenetic risk groups poor and very poor according to the Revised International Prognostic Scoring System (IPSS-R) were found in 11 *SF3B1*nent but in none of *SF3B1*ent cases (Supplementary Fig. S5B).

**Fig. 4:**
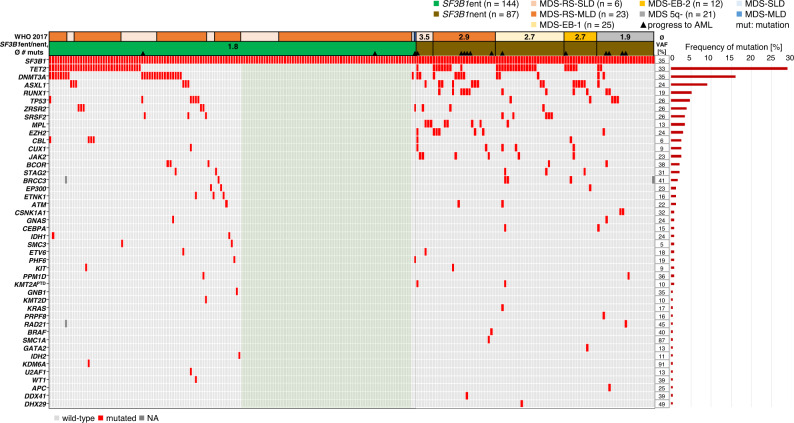
Molecular characterization of *SF3B1*^mut^ MDS patients. Illustration of all 231 samples, each column represents one patient. Genes (gray: wild-type; red: mutated) as well as the WHO entity (incl. *SF3B1*ent) are given for each patient. Light green: *SF3B1*ent patients with isolated *SF3B1* mutation; VAF variant allelic frequency.

Within *SF3B1*ent 47% (67/144) did not harbor any additional mutation in 73 analyzed genes resulting in an average of 1.8 mutations (including *SF3B1*) in this group (Fig. 4), while 53% (77/144) harbored one to four additional mutations. *SF3B1*nent patients showed on average 2.6 mutations (MDS with isolated del(5q): 1.9; MDS-EB: 2.7; MDS-RS: 3.1; Fig. 4). Although *SF3B1*ent samples showed in total few mutations, additional mutations (if present) were detected in 27 different genes (Supplementary Fig. S6A). Additional mutations in *SF3B1*nent samples were found in 9 to 20 different genes depending on the respective entity (Supplementary Fig. S6B–F).

The most frequent additional mutations in all *SF3B1*^mut^ patients were *TET2* (29%), *DNMT3A* (16%) and *ASXL1* (9%) (Fig. 4; Supplementary Fig. S7A, B). The mutational frequencies of *RUNX1, MPL*, *EZH2,* and *JAK2* in the total *SF3B1*^mut^ cohort were 5% (*RUNX1*) and 3% (*MPL, EZH2*, *JAK2*) and were present due to the entity criteria only in *SF3B1*nent in 12, 8, 7 and 6 cases, respectively. Of note, compared to *SF3B1* wild-type samples mutations in *ASXL1, RUNX1, TP53, ZRSR2, SRSF2* and *STAG2* were significantly less frequent in *SF3B1*^mut^ patients, while *DNMT3A* mutations were more frequent (Supplementary Fig. S7A). Interestingly, within *SF3B1*^mut^ samples *TP53* mutations (*n* = 11) were most frequently seen within MDS 5q- (3/21, 14%). However, mutated *TP53* was also seen in *SF3B1*ent (6/144; 4%), MDS-EB-1 (1/25; 4%) and MDS-EB-2 (1/12; 8%) (Supplementary Fig. S7B). Notably, 82% (9/11) of *TP53* mutations were monoallelic events. In two samples (MDS-EB-1/2) both a mutation and deletion were detected affecting the *TP53* gene (biallelic inactivation). In 17 *SF3B1*^mut^ samples, additional spliceosome mutations were found, namely *ZRSR2* (*n* = 9) and *SRSF2* (*n* = 8) (Fig. 4; Supplementary Figs. S6, S7). In *SF3B1*ent patients spliceosome mutations were found in 9 cases (*ZRSR2*: *n* = 6, mean VAF: 27% vs. 26% of *SF3B1*; *SRSF2*: *n* = 3, mean VAF: 16% vs. 47% of *SF3B1*), whereas within *SF3B1*nent 8 samples showed additional *ZRSR2* (*n* = 3, mean VAF: 26% vs. 37% of *SF3B1*) or *SRSF2* (*n* = 5, mean VAF: 31% vs. 29% of *SF3B1*) mutations (Supplementary Fig. S8). Interestingly, additional spliceosome mutations were not detected in MDS 5q- (Fig. 4; Supplementary Fig. S7). In 5/17 (29%) cases the *SF3B1* VAF was lower than the VAF of additional spliceosome mutations (*SRSF2*: *n* = 2, all MDS-EB-1; *ZRSR2*: *n* = 3, all *SF3B1*ent; Supplementary Fig. S8). In 4 of those samples the *SF3B1* VAF was lower than 15% and therefore accounted to the 13 samples of the entire MDS cohort showing only one *SF3B1* mutation with a low VAF (<15%; Fig. 3B). Thus, in 11/13 patients with a low *SF3B1* VAF either deletions on chromosome 5 (*n* = 5), additional spliceosome (*n* = 4) or *TP53* mutations (*n* = 2) were identified at MDS diagnosis.

### Prognostic impact of additional aberrations in SF3B1^mut^ MDS

Next, we analyzed the prognostic contribution of additional gene mutations and other risk factors to OS in *SF3B1*^mut^ patients. Within all *SF3B1*^mut^ patients the number of mutations showed a significant impact on OS (Supplementary Fig. S9A; *p* = 0.040). However, within *SF3B1*ent cases, OS was not affected by the presence of additional mutations (Supplementary Fig. S9B). In univariate analyses bone marrow blasts <5% was a good prognostic marker (hazard ratio HR: 0.616; *p* = 0.033), while *RUNX1* mutations (HR: 4.347; *p* < 0.001), *ASXL1* mutations (HR: 1.836, *p* = 0.023) and del(5q) (HR: 1.977; *p* = 0.008) were poor prognostic markers (Table 2). Of note, the poor prognostic impact on OS of complex karyotypes did not reach statistical significance (*p* = 0.063) presumably due to the small samples size (*n* = 7). Further, del(5q) was not restricted to isolated del(5q) cases but comprised all cases with deletions on chromosome 5, and thus included also cases with complex karyotypes. In multivariate analysis only *RUNX1* mutations (HR: 3.581; *p* < 0.001) and del(5q) (HR: 2.146; *p* = 0.003) were independent prognostic factors. Patients with *SF3B1*^mut^ having either del(5q) or *RUNX1* mutations (n = 31) showed shorter OS compared to *SF3B1*^mut^ patients not having these abnormalities (*n* = 200; median OS: 43 vs. 88 months; *p* < 0.001; Supplementary Fig. S9C).

**Table 2:** Cox proportional hazards ratio analyses of variables in *SF3B1* mutated MDS prognostic of OS.

Risk factor	Hazard ratio (HR)	95% CI	*P*
Univariate analysis			
Sex	1.080	0.764–1.528	0.663
* SF3B1* VAF, < 15% vs. ≥15%	0.705	0.345–1.443	0.339
Bone marrow blast count, <5% vs. ≥5%	0.616	0.395–0.961	**0.033**
Bone marrow blast count, <10% vs. ≥10%	1.901	0.605–5.980	0.272
* RUNX1*	4.347	2.325–8.128	**<0.001**
* EZH2*	1.381	0.564–3.381	0.480
* ASXL1*	1.836	1.085–3.105	**0.023**
* DNMT3A*	0.920	0.582–1.454	0.720
* TET2*	0.979	0.682–1.405	0.907
* JAK2*	1.115	0.411–3.022	0.831
* MPL*	1.520	0.709–3.257	0.282
* TP53*	1.401	0.652–3.008	0.387
del(5q)	1.977	1.198–3.262	**0.008**
Complex karyotype (≥3 abnormalities)	2.986	0.944–9.448	0.063
* MECOM* rearrangement	0.486	0.069–3.556	0.497
Other cytogenetic abnormalities	1.428	0.930–2.194	0.104
Multivariate analysis			
Bone marrow blast count, <5% vs. ≥5%	0.693	0.418–1.148	0.154
* RUNX1*	3.581	1.769–7.249	**<0.001**
* ASXL1*	1.157	0.618–2.164	0.649
del(5q)	2.146	1.289–3.574	**0.003**

*OS* overall survival, *CI* confidence interval, *VAF* variant allelic frequency.

*p*-values in bold: statistical significance (*p* < 0.05).

### Molecular genetics of SF3B1^mut^ patients transforming to AML

**Fig. 5:**
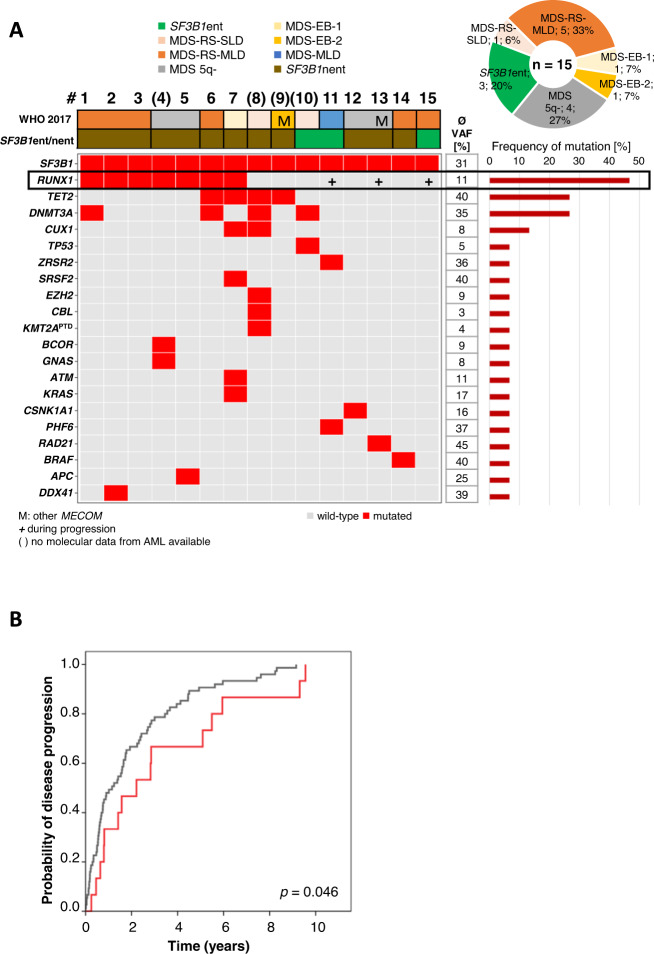
Genetics of MDS patients with mutated *SF3B1* progressing to AML. **A** Molecular characterization of *SF3B1*^mut^ patients progressing to AML at MDS stage (*n* = 15). Each column represents one patient, numbered 1–15. Number in brackets indicate that molecular data at AML stage is not available. Genes (gray: wild-type; red: mutated), WHO 2017 entities and *SF3B1*ent/nent are given for each patient. ent entity, nent non-entity, VAF variant allelic frequency. **B** Cumulative incidence of AML transformation of *SF3B1* mutated (*n* = 15; red) vs. wild-type (*n* = 75; gray) patients.

Of *SF3B1*^mut^ patients 7% (15/231) progressed to AML compared to 15% (75/503) of *SF3B1* wild-type patients (median follow-up: 9.3 years; Fig. 5A). In addition, time to AML was shorter in *SF3B1* wild-type compared to *SF3B1*^mut^ patients (median: 14 vs. 27 months, *p* = 0.046; Fig. 5B). Notably, an AML transformation rate of 14% (12/87) was seen in *SF3B1*nent and 2% (3/144) in *SF3B1*ent (median follow-up: 122 and 112 months; Fig. 5A). A trend for a longer time to AML transformation was observed for *SF3B1*ent compared to *SF3B1*nent, however not reaching statistical significance (71 vs. 17 months, *p* = 0.0825). Eleven of 15 *SF3B1*^mut^ MDS cases were also analyzed for the presence of molecular mutations at their diagnosis of AML (Fig. 5A, further details are provided in the supplement). Regarding the prognostic contribution of additional gene mutations and other risk factors to AML transformation in *SF3B1*^mut^ patients univariate analyses revealed bone marrow blasts <5% to be associated with lower risk (hazard ratio HR: 0.097; *p* = 0.021) and *RUNX1* mutations (HR: 3.518; *p* = 0.05) with higher risk for AML transformation (Supplementary Table S3).

## Discussion

*SF3B1* mutations are frequently detected within MDS and associated with favorable prognosis [5–7]. In our WGS-based cohort of 734 MDS patients we identified 231/734 (31%) cases with *SF3B1* mutations verifying known hotspots in K700, K666 and H662 [4, 7, 8, 20, 21] and confirming a heterozygous *SF3B1* mutation status with high median VAFs (35%) across all entities. VAFs >30% were observed in 77% of *SF3B1*^mut^ samples. *SF3B1* mutations persisted over the entire disease courses in many AML-transforming patients supporting that *SF3B1* mutations are early events in MDS. However, *SF3B1*^mut^ samples with low VAFs (<15%) were mainly found in *SF3B1* non-entity cases showing excess blasts, del(5q) or *TP53* mutations but also in two *SF3B1*ent samples harboring other spliceosome mutations. In line with previous reports, *SF3B1* mutations were predominantly found in MDS-RS-SLD/MLD supporting the association of *SF3B1* with RS [7, 10]. Moreover, we confirmed that *SF3B1* mutations in MDS were favorable with regard to OS and AML transformation [7, 10, 11].

Recently, the IWG-PM suggested MDS with mutated *SF3B1* as a distinct entity [11]. In this study, we evaluated the IWG-PM proposed *SF3B1* entity criteria. We confirmed the favorable clinical outcome of *SF3B1* entity similar to recently published studies [21, 22]. Additionally, in line with Komrokji et al. we observed a significantly longer OS of *SF3B1* entity patients compared to *SF3B1* non-entity patients, in contrast to Venable et al. who did not observe significant differences in OS between *SF3B1*ent and *SF3B1*nent presumably due to the small cohort size [21].

In contrast to Malcovati et al. [11], we observed that *SF3B1* mutations were associated with significantly shorter OS within MDS 5q- concordant with previous reports [19, 22, 23] highlighting the adverse prognostic impact of mutated *SF3B1* within this entity. Within our *SF3B1*^mut^ cohort, only 5% (11/231; all *SF3B1*nent) had poor or very poor cytogenetic risk groups concordant with a previous report [11] adding to the reasons for the favorable prognosis of *SF3B1* mutations. In this line, the lately published IPSS-M, a unique risk score, improves the risk stratification of MDS patients by including molecular genetics into their model [24], in contrast to the IPSS-R, which considers only morphological features and cytogenetics [25]. The IPSS-M model further incorporates *SF3B1* mutations with different weights depending on co-abnormalities (i.e. isolated del(5q) or *BCOR, BCORL1, RUNX1, NRAS, STAG2, SRSF2* mutations).

With regard to the mutational landscape of *SF3B1*^mut^ cases the most frequent additional mutations were *DNMT3A, TET2*, and *ASXL1* (DTA) similar to previous reports showing that epigenetic and histone modifiers are commonly mutated in MDS, but also in aging individuals [5, 21, 26–28]. The number of additional mutations significantly impacted on OS in all *SF3B1*^mut^ patients. Within *SF3B1*ent the number of co-mutations did not affect OS as shown in IWG-PM results [11], however the number of co-mutations was low compared to *SF3B1*nent cases.

In line with previous studies [5, 10], we confirmed that progression to AML occurs at a relatively low frequency in *SF3B1*^mut^ patients (7%; 15/231). Furthermore, AML transformation was less frequent in *SF3B1*ent compared to *SF3B1*nent (2% vs. 14%). Progression of MDS to AML is suggested to be driven by cooperating genetic lesions [28, 29]. In this regard, we found that AML-transforming patients harbored on average more mutations than non-progressing patients (3.2 vs. 2.0). We further demonstrated that at MDS diagnosis 47% (7/15) of AML-transforming patients showed *RUNX1* mutations, significantly more frequent in AML-transforming compared to non-transforming patients. Moreover, during disease progression chromosomal aberrations were gained in two cases whereas most frequently *RUNX1* mutations were acquired (*n* = 3) highlighting the role as potential driver gene and confirming the strong adverse prognostic value of *RUNX1* mutations [11] associated with worse OS and a higher AML transformation rate within *SF3B1*^mut^ patients as also shown by Komrokji et al. [22].

Recently, the 5th edition of WHO and the ICC introduced MDS with mutated *SF3B1* as a new entity [2, 12]. The entity criteria proposed by the WHO 2022 and ICC mainly follow those suggested by the IWG-PM but differ in excluding mutations. Further, it is stated that the diagnostic criteria of MDS 5q- remain and that an *SF3B1* mutation does not per se override this diagnosis. This is supported by our data as *SF3B1* mutations show a negative impact on OS in MDS 5q- and do not seem to be the defining mutation in this setting, as suggested by the frequently low *SF3B1* VAF. In contrast to the IWG-PM proposal, both WHO 2022 and ICC guidelines exclude biallelic *TP53* inactivations from the *SF3B1* entity. In our *SF3B1*^mut^ cohort, only two samples (2/231; <1%) harbored biallelic *TP53* inactivations (both with blast count >5%). Concordant with the IWG-PM, ICC also excludes *RUNX1* mutations from the *SF3B1* entity supported by our data showing *RUNX1* mutations as independent negative prognostic factors for OS and AML transformation.

In our univariate analysis, we confirmed the negative prognostic impact of del(5q) on OS of *SF3B1*^mut^ cases and additionally found a negative impact of blast count >5% as well as *RUNX1* and *ASXL1* mutations. However, our multivariate analysis could not confirm the independent prognostic impact of blast count >5%, but showed del(5q) and *RUNX1* mutations as independent prognostic markers. Thus, based on our data the threshold of <5%, which is used by IWG-PM, ICC and WHO 2022, is not required if presence of del(5q) and *RUNX1* mutation are exclusion criteria for the *SF3B1* entity. Of note, studies from Malcovati et al. showed a significant impact of excess blasts on the survival of *SF3B1*^mut^ patients [10, 11], however, *RUNX1* mutations were not included in their multivariate analysis.

In conclusion, *SF3B1* mutations are associated with good clinical outcome. Patients fulfilling the criteria of the *SF3B1* entity proposed by the IWG-PM show an even better prognosis (longer OS, lower AML transformation rate). Our data suggest that the identification of the good prognostic subset within *SF3B1*^mut^ patients can be achieved by excluding only cases with del(5q) and/or *RUNX1* mutations, however completely independent of blast count.

## Supplementary information

Supplementary Information

## Data Availability

The datasets generated during and/or analyzed during the current study are available from the corresponding author on reasonable request.
