# Supplementary material for: SF3B1 mutated MDS: Blast count, genetic co-abnormalities and their impact on classification and prognosis
Source: Leukemia. 2022 Oct 19;36(12):2894–902. doi: 10.1038/s41375-022-01728-5 (PMC9712089; doi:10.1038/s41375-022-01728-5)
Supplement: Supplementary file 1 — Supplementary Information [file 41375_2022_1728_MOESM1_ESM.pdf]

Supplementary Material

Supplementary Methods

Mutational analysis

The following 73 myeloid genes were analyzed within the study: *ASXL1, APC, ASXL2, ATM, ATRX, BCOR, BCORL1, BRAF, BRCC3, CALR, CBL, CDH23, CDKN2A, CEBPA, CREBBP, CSF3R, CSNK1A1, CTCF, CUX1, DDX41, DDX54, DHX29, DNMT3A, EP300, ETNK1, ETV6, EZH2, FANCL, FBXW7, FLT3*-TKD*, GATA1, GATA2, GNAS, GNB1, IDH1, IDH2, JAK2, KDM5A, KDM6A, KIT, KMT2D, KRAS, MPL, MYC, NF1, NOTCH1, NPM1, NRAS, PHF6, PIGA, PPM1D, PRPF8, PTPN11, RAD21, RB1, RUNX1, SETBP1, SF1, SF3A1, SF3B1, SH2B3, SMC1A, SMC3, SRSF2, STAG2, SUZ12, TET2, TP53, U2AF1, U2AF2, WT1, ZBTB7A, ZRSR2*.

Results

Changes in MDS diagnoses according to WHO 2022 and ICC

Several changes in MDS diagnoses were seen comparing the different MDS-*SF3B1* entity criteria proposed by either the IWG-PM with underlying WHO 2017, the WHO 2022 or ICC (Figure 2C). In this regard, 18 former *SF3B1*nent samples qualified for MDS-*SF3B1* based on WHO 2022 (*EZH2:* n = 5; *RUNX1*: n = 6; *JAK2 or MPL* without thrombocytosis: n = 7). Regarding ICC, two *SF3B1*ent cases were excluded from the MDS-*SF3B1* entity due to low *SF3B1* VAFs (<10%). Cases with mutated *EZH2* (n = 5) and mutated *JAK2 or MPL* without thrombocytosis (n = 7) were defined as MDS-*SF3B1* in line with WHO 2022.

From *SF3B1*nent cases, 4 samples with *JAK2* or *MPL* mutations showed thrombocytoses and were thus classified as MDS/MPN-*SF3B1*-T or MDS/MPN-T-*SF3B1*, based on WHO 2022 or ICC, respectively. As two other *SF3B1*nent samples

harbored biallelic *TP53* inactivations, they were categorized into the corresponding new entities, MDS with biallelic *TP53* inactivation regarding WHO 2022 or MDS with mutated *TP53* when considering ICC. In addition, 3 *SF3B1*nent samples (blasts <10%: n = 2) had a *MECOM* rearrangement therefore being assigned to AML according to WHO 2022. However, those cases would not be classified as AML based on ICC, as at least 10% blasts and certain partner genes of *MECOM* are required for diagnosing AML with other *MECOM*. Of note, 11 former MDS-EB-2 samples would be grouped into the new MDS/AML category.

Following WHO 2022 guidelines, MDS-*SF3B1* patients showed a median OS of 95 months (Suppl. Figure S1C).

Genetics of SF3B1^{mut} patients progressing to AML at MDS diagnosis

SF3B1^{mut} patients progressing to AML showed on average 3.2 mutations while non-progressing *SF3B1*^{mut} patients harbored on average 2.0 mutations (Figure 4, 5). The number of mutations significantly impacted the time to AML transformation within all AML-transforming patients (n = 90), but not within *SF3B1*^{mut} AML-transforming patients (n = 15; Suppl. Figure S10A, B). The most frequent additional mutation in AML-transforming *SF3B1*^{mut} patients was *RUNX1* detected in 47% (7/15; Figure 5A). Notably, time to AML was shorter in *RUNX1* mutated compared to wild-type patients within all AML-transforming MDS patients (median: 10 vs. 19 months, p = 0.030; Suppl. Figure S10C) and also within *SF3B1*^{mut} AML-transforming patients (median: 10 vs. 34 months, p = 0.038; Suppl. Figure S10D). In non-progressing *SF3B1*^{mut} patients the frequency of *RUNX1* mutations at MDS diagnosis was significantly lower (2%; 5/216; p < 0.001; Suppl. Figure S10E). Following this, 58% (7/12) of all *SF3B1/RUNX1* mutated patients showed AML transformations. The second most frequent additional mutations at MDS diagnosis in AML-transforming *SF3B1*^{mut} patients were *DNMT3A*

and *TET2* mutations (each 27%; 4/15; Figure 5A) showing similar mutational frequencies in non-progressing *SF3B1*^{mut} patients (*DNMT3A*: 15%, 33/216; *TET2*: 29%, 63/216; Figure 4; Suppl. Figure S10E) and no effect on AML-transformation in MDS (Suppl. Figure S10F, G). Furthermore, three AML-transforming patients showed a *SF3B1* VAF <15% at MDS diagnosis (Figure 5A: #7, 11, 12). In two of these additional spliceosome mutations - *SRSF2* (n = 1; VAF: 40%) or *ZRSF2* (n = 1; VAF: 38%) - were identified at MDS diagnosis while the third sample (MDS 5q-; #12) harbored a *CSNK1A1* mutation (VAF: 16%). Regarding cytogenetics, in total 4/15 (27%) patients showing AML-progression were MDS 5q-, while two others harbored *MECOM* rearrangements (-r) when MDS was diagnosed (Figure 5A: #9, 14; Suppl. Figure S10H). Thus, 2/3 (67%) *SF3B1*^{mut} MDS with *MECOM*-r progressed to AML, one (Figure 5A: #9, MDS-EB-2) after three and the other (Figure 5A: #14, MDS-RS-MLD) after 27 months.

Genetics of SF3B1^{mut} patients progressing to AML at AML stage

During disease progression two *SF3B1*ent (Figure 5A: #11, 15) and one *SF3B1*nent (Figure 5A: #13, MDS 5q-) patients gained *RUNX1* mutations out of which one (#15) additionally gained a 7q deletion (Suppl. Figure S11, S12). The gain of other chromosomal aberrations (i.e. 17p deletion and *MECOM*-r) were detected in one (MDS 5q-) patient during AML transformation (Figure 5A: #12, Suppl. Figure S12). Another patient gained three mutations other than *RUNX1* when progressing to AML (Figure 5A: #14, Suppl. Figure S12).

SF3B1 and RUNX1 mutations during AML transformation

Focusing on the *SF3B1* levels during disease progression, in 12 of 15 (80%) cases a high VAF of *SF3B1* (27-44%) was observed at MDS diagnosis (Suppl. Figure S13: red). Here, in 8 patients the *SF3B1* levels persisted over the entire disease courses

(Suppl. Figure S11: #1, 2, 3, 5, 6, 13, 15; Suppl. Figure S12: #14; no AML data in 4/12). In one of those patients, relapsing during follow-up, the *SF3B1* mutation re-occurred after stem cell transplantation (Figure 5A/ Suppl. Figure S11: #3; Suppl. Figure S13: dark red). For 3/15 (20%) patients low *SF3B1* VAFs (<15%) were detected at MDS diagnosis (Suppl. Figure S13: light brown; Suppl. Figure S12: #7, 11, 12; Figure 3B). Of those, in two cases the VAFs of *SF3B1* decreased during AML transformation while the VAFs of additional other spliceosome mutations increased (Suppl. Figure S12: #7, 11). In the third case with a low *SF3B1* VAF, a MDS 5q- patient, the VAF of *SF3B1* increased while gaining several chromosomal aberrations during disease course (Figure 3B; Figure 5A/ Suppl. Figure S12: #12).

With regard to *RUNX1* mutations, within the 15 *SF3B1* mutated patients progressing to AML 67% (10/15) either showed additional *RUNX1* mutations (n = 7) at MDS diagnosis or gained *RUNX1* (n = 3) mutations during disease progression (Figure 5A; Suppl. Figure S11). In all 7 cases with mutated *SF3B1* and *RUNX1* at the time of MDS diagnosis the VAF of *RUNX1* was lower than the VAF of *SF3B1* at any time, also during disease courses (Figure 5: #1-7; Suppl. Figure S11: #1-6; Suppl. Figure S12: #7). At AML state the VAF of *SF3B1* exceeded the VAF of *RUNX1* in 6/10 cases (Suppl. Figure S11: #1, 2, 3, 5, 13, 15; Figure 5A: #4 no AML data). In 3 patients the VAF of *RUNX1* was higher than the one of *SF3B1* at the time of AML diagnosis (Suppl. Figure S11: #6, Suppl. Figure S12: #7, 11). In one of those patients a CN-LOH overlapping *RUNX1* (Suppl. Figure S11: #6) was found at AML diagnosis whereas the other two patients showed additional spliceosome mutations (*SRSF2* or *ZRSR2)* during the entire disease course in the presence of a low *SF3B1* VAF (Figure 5A, Suppl. Figure S12: #7, 11).

Of the remaining 5 patients being *RUNX1* negative through the complete follow-up, three patients either harbored chromosomal aberrations at the time of MDS diagnosis or gained one during disease progression (Figure 5A, Suppl. Figure S12: #9,12,14; Suppl. Figure S10H). In the other *RUNX1* negative patients, in one more than 5 mutations and in the other a *TP53* mutation were detected at MDS diagnosis (Figure 5A: #8,10; Suppl. Figure S10H).

With regard to the *SF3B1*ent samples progressing to AML (n = 3; Figure 5A, D: green), one patient (Figure 5A: #10) harbored additional *TP53* mutation (VAF: 5%), one (Figure 5A/ Suppl. Figure S11: #15) gained a *RUNX1* mutation and a chromosome 7q deletion during AML transformation and the other gained two different *RUNX1* mutations in the presence of low *SF3B1* and high *ZRSR2* levels (Figure 5A/ Suppl. Figure S12: #11).

AML progression in *SF3B1*^{mut} MDS patients was determined by the entire genomic landscape, i.e. number and VAF of additional mutations at MDS diagnosis, presence and gain of chromosomal aberrations or certain mutations during AML transformation indicating *RUNX1* as strong driver (Suppl. Figure S10H, S11, S12). Samples with a low *SF3B1* VAF were mostly found in *SF3B1*nent including prognostic unfavorable entities. In these cases, the prognosis seems to be determined by other spliceosome or genetic alterations already present at MDS stage or gained during disease course.

Supplementary Tables and Figures

Table S1. Classification and entity criteria of MDS with *SF3B1* mutations

	**Revised 4th edition of WHO + IWG-PM [A]**	**5th edition of WHO [B]**	**ICC [C]**
**Criteria**	MDS with mutated *SF3B1*	MDS with low blasts and *SF3B1* mutation	MDS with mutated *SF3B1*
Cytopenia	≥ 1	≥ 1	≥ 1
Dysplasia	≥ 1	≥ 1	Not required
Blasts	< 5% BM; < 1% PB	< 5% BM; < 2% PB	< 5% BM; < 2% PB
Cytogenetics	Absence of 5q deletion, monosomy 7, inv(3) or abnormal 3q26, complex karyotype	Absence of 5q deletion, monosomy 7, or complex karyotype **	Absence of isolated del(5q), -7/del(7q), abn3q26.2, or complex
Mutations	*SF3B1* without *RUNX1 and/or EZH2* *	*SF3B1* without biallelic *TP53* **	*SF3B1* (VAF ≥ 10%) without *RUNX1* or multi-hit *TP53*
RS	Not required	Not required	Not required

[A] Malcovati *et al.,* Blood, 2020; [B] Khoury *et al.,* Leukemia, 2022; [C] Arber *et al.,* Blood, 2022

**JAK2*V617F, *CALR*, or *MPL* mutations strongly support the diagnosis of MDS/MPN-RS-T.

**Excluding AML-defining genetic abnormalities

Table S2. Overview of patients receiving allogeneic stem cell transplantation

**WHO 2017 Diagnosis**	**Number of samples, n**	**Allogeneic STC**	***SF3B1*mut**	***SF3B1*wt**
MDS-SLD	22	0	0	0
MDS-MLD	105	4	0	4
MDS-RS-SLD	51	1	0	1
MDS-RS-MLD	149	7	3	4
MDS 5q-	107	0	0	4
MDS-EB-1	149	19	3	16
MDS-EB-2	151	19	3	16
**MDS total**	**734**	**54**	**9**	**45**

SCT: stem cell transplantation; mut: mutated; wt: wild-type

Table S3. Cox proportional hazards ratio analyses of variables in *SF3B1* mutated MDS prognostic of AML transformation

Risk factor	Hazard ratio (HR)	95% CI	*P*
Univariate analysis			
Sex	1.454	0.490 - 4.313	0.5
SF3B1 VAF, <15% vs. ≥15%	2.591	0.565 - 11.887	0.221
Bone marrow blast count, <5% vs. ≥5%	0.097	0.013 - 0.702	**0.021**
RUNX1	3.518	1.001 - 12.367	**0.05**
EZH2	0.356	0.044 - 2.905	0.335
DNMT3A	0.834	0.247 - 2.809	0.769
TET2	1.645	0.491 - 5.506	0.419
TP53	0.499	0.063 - 3.960	0.51
Del(5q)	1.546	0.455 - 5.248	0.485
MECOM rearrangement	3.059	0.612 - 15.285	0.173
Other cytogenetic abnormalities	0.962	0.121 - 7.675	0.971
Number of mutations, ≤2 vs. >2	0.650	0.204 - 2.069	0.466
Multivariate analysis			
Bone marrow blast count, <5% vs. ≥5%	0.143	0.020 - 1.046	0.055
RUNX1	3.032	0.820 - 11.206	0.096

CI: confidence interval; VAF: variant allelic frequency

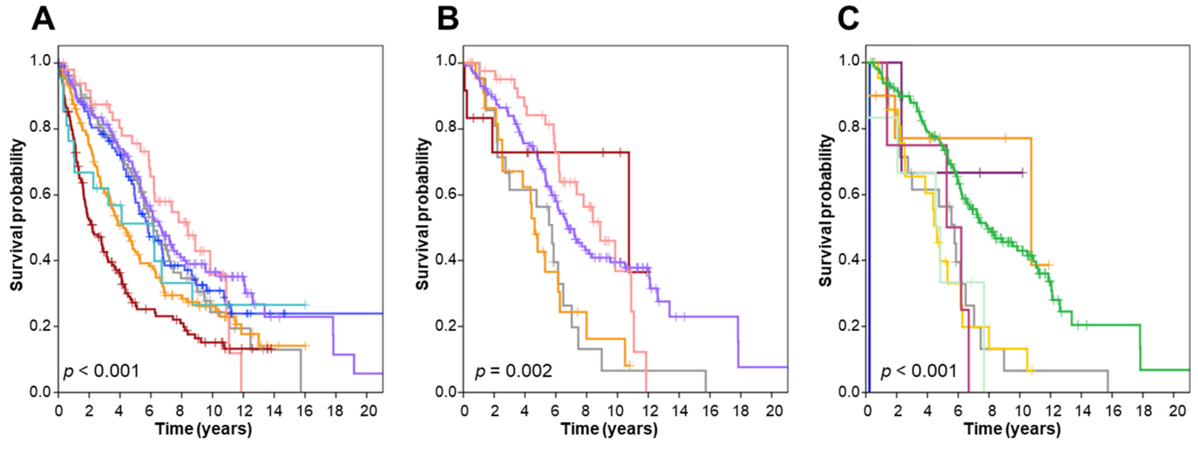

Supplementary Figure S1: Overall survival (OS) of MDS patients. (A) OS of all MDS cases according to WHO 2017 entity (pink: MDS-RS-SLD, n = 51; purple: MDS-RS-MLD, n = 149; grey: MDS 5q-, n = 107; dark blue: MDS-MLD, n = 105; light blue: MDS-SLD, n = 22; orange: MDS-EB-1, n = 149; red: MDS-EB-2, n = 151) ($p < 0.001$). (B) OS of *SF3B1* mutated MDS according to WHO 2017 entity (pink: MDS-RS-SLD, n = 43; purple: MDS-RS-MLD, n = 128; grey: MDS 5q-, n = 21; orange: MDS-EB-1, n =

25; red: MDS-EB-2, n = 12) (*p* = 0.002). MDS-SLD and MDS-MLD patients are not shown due to small sample size (n = 1 each). (C) OS of *SF3B1* mutated MDS according to WHO 2022 entity (green: MDS-*SF3B1*, n = 162; light green: MDS-LB, n = 6; grey: MDS-5q, n = 21; yellow: MDS-IB1, n = 23; orange: MDS-IB2, n = 10; dark purple: AML with *MECOM*-r, n = 3; magenta red: MDS/MPN-*SF3B1*-T, n = 4; blue: MDS-bi*TP53*, n = 2) (*p* < 0.001).

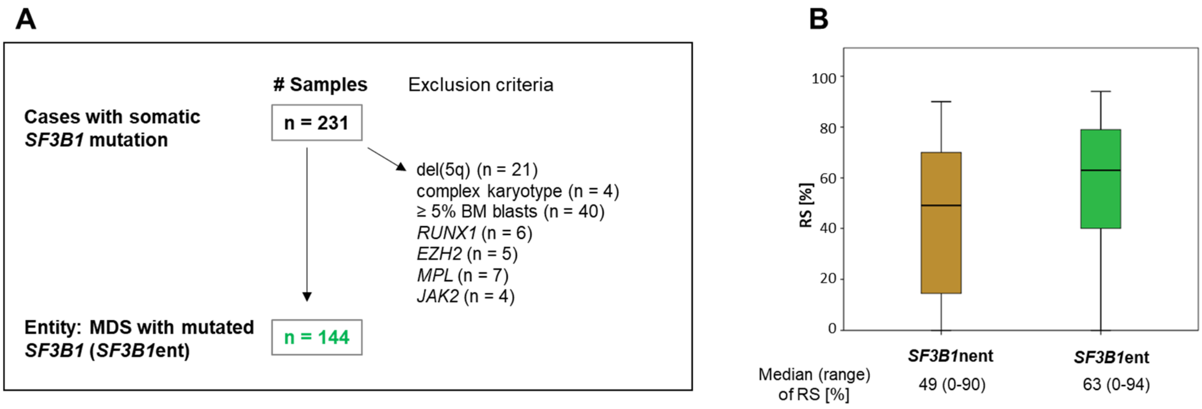

Supplementary Figure S2: Classification into *SF3B1* entity and ring sideroblasts of *SF3B1* mutated samples. (A) Number of samples fulfilling the criteria for the proposed *SF3B1* entity (*SF3B1*ent); BM: bone marrow. (B) Boxplot of ring sideroblasts (RS) of samples from *SF3B1*ent or non-*SF3B1*ent (*SF3B1*nent). The central horizontal line within each box indicated the median. The interquartile range is indicated by the top and bottom edges of each box.

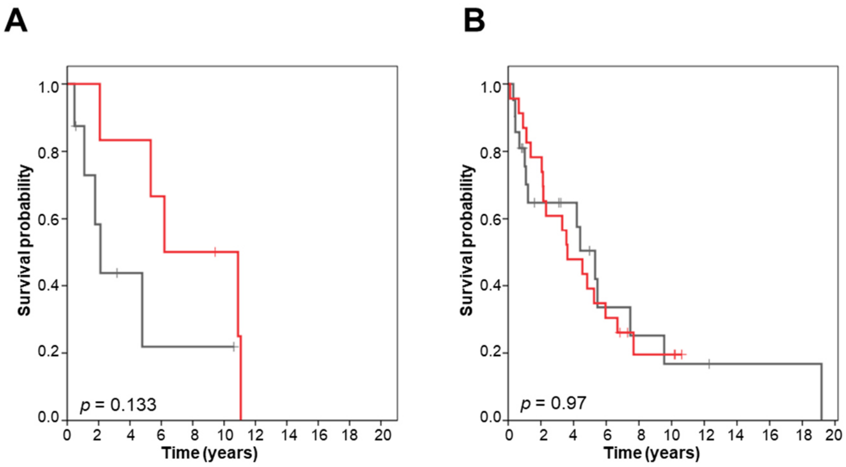

Supplementary Figure S3: OS of MDS-RS-SLD/MLD after exclusion of cases fulfilling *SF3B1* entity criteria. (A) OS of MDS-RS-SLD according to *SF3B1* mutation status (mutated/ *SF3B1*nent: n = 6, red; wild-type: n = 8, grey) (p = 0.133). (B) OS of MDS-RS-MLD according to *SF3B1* mutation status (mutated/*SF3B1*nent: n = 23, red; wild-type, n = 21, grey) (p = 0.97). OS: overall survival.

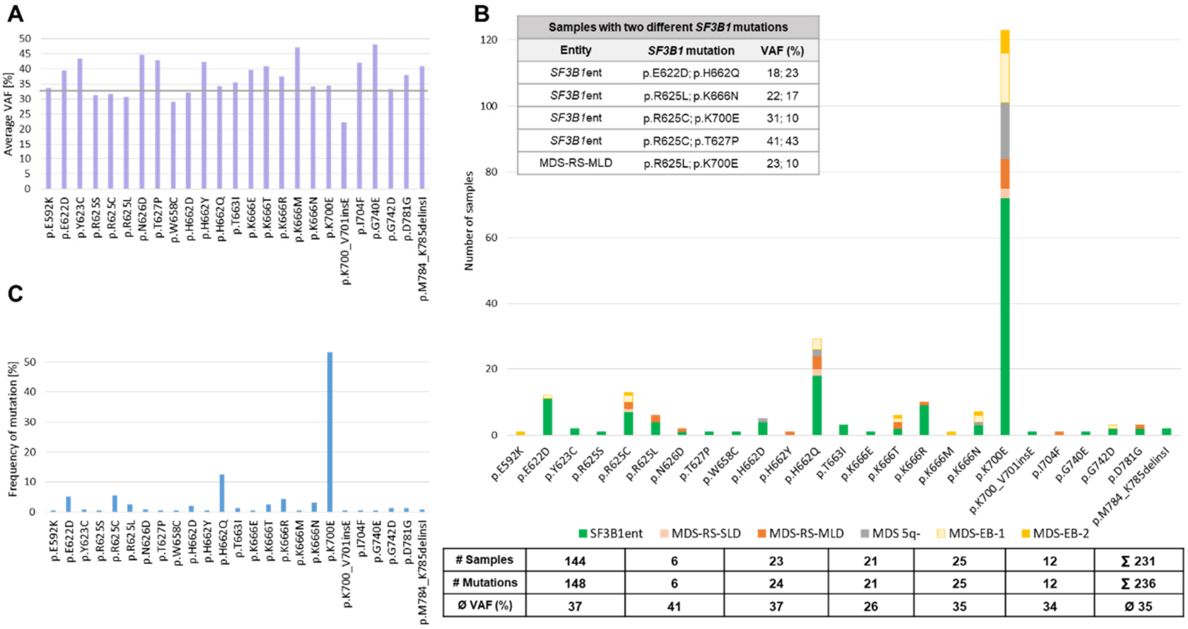

Samples with two different *SF3B1* mutations		
**Entity**	***SF3B1* mutation**	**VAF (%)**
*SF3B1*ent	p.E622D; p.H662Q	18; 23
*SF3B1*ent	p.R625L; p.K666N	22; 17
*SF3B1*ent	p.R625C; p.K700E	31; 10
*SF3B1*ent	p.R625C; p.T627P	41; 43
MDS-RS-MLD	p.R625L; p.K700E	23; 10

	SF3B1ent	MDS-RS-SLD	MDS-RS-MLD	MDS 5q-	MDS-EB-1	MDS-EB-2	
**# Samples**	**144**	**6**	**23**	**21**	**25**	**12**	**∑ 231**
**# Mutations**	**148**	**6**	**24**	**21**	**25**	**12**	**∑ 236**
**Ø VAF (%)**	**37**	**41**	**37**	**26**	**35**	**34**	**Ø 35**

Supplementary Figure S4: Variety of *SF3B1* mutations in MDS. (A) Average variant allelic frequency (VAF) of different *SF3B1* mutations (n = 263). (B) Distribution of detected *SF3B1* mutations among MDS subgroups. Samples with two different *SF3B1* mutations are shown in the Table. ∑: in total; (C) Frequency of *SF3B1* mutations within all *SF3B1* mutated samples (n = 231).

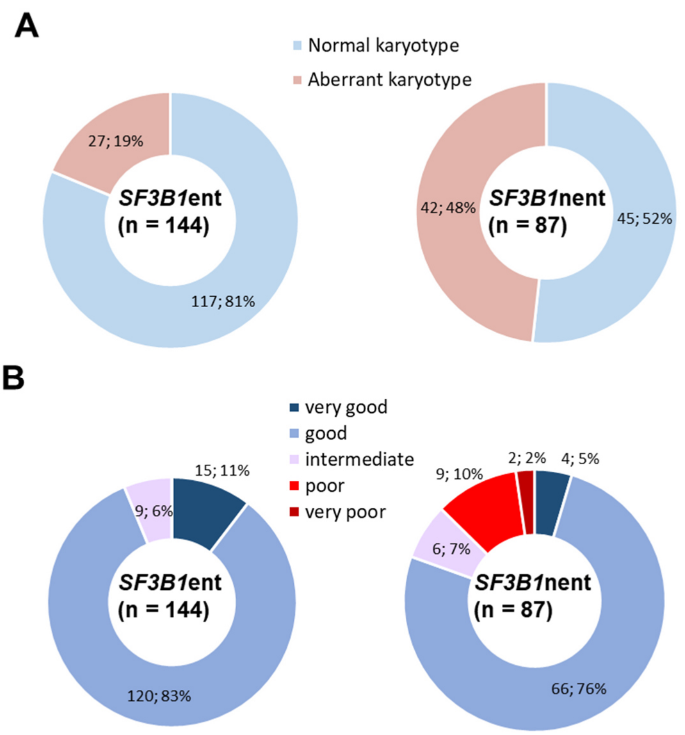

Supplementary Figure S5: Cytogenetics of *SF3B1* mutated samples Karyotypes (A) and cytogenetic risk groups (B) of *SF3B1* mutated samples comparing *SF3B1*ent (left) vs. *SF3B1*nent (right).

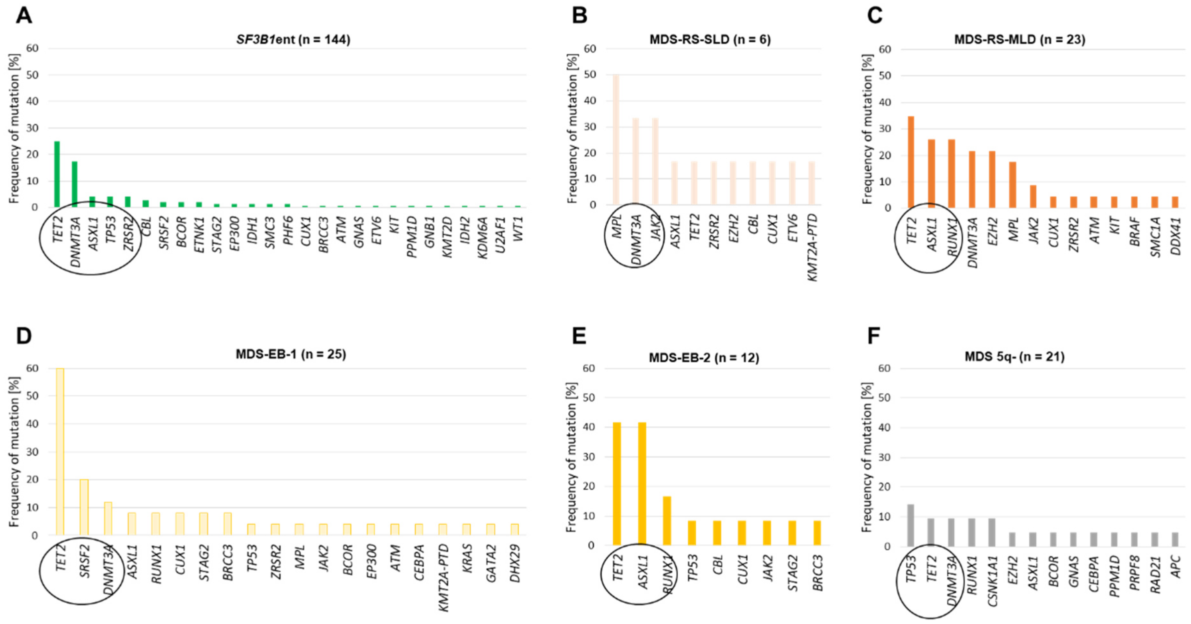

Supplementary Figure S6: Additional gene mutations in *SF3B1* mutated patients. Frequency of additional gene mutations within *SF3B1*ent (A), MDS-RS-SLD (B), MDS-RS-MLD (C), MDS-EB-1 (D), MDS-EB-2 (E) and MDS 5q- (F). The 3 most frequent mutations within each entity are marked with circles.

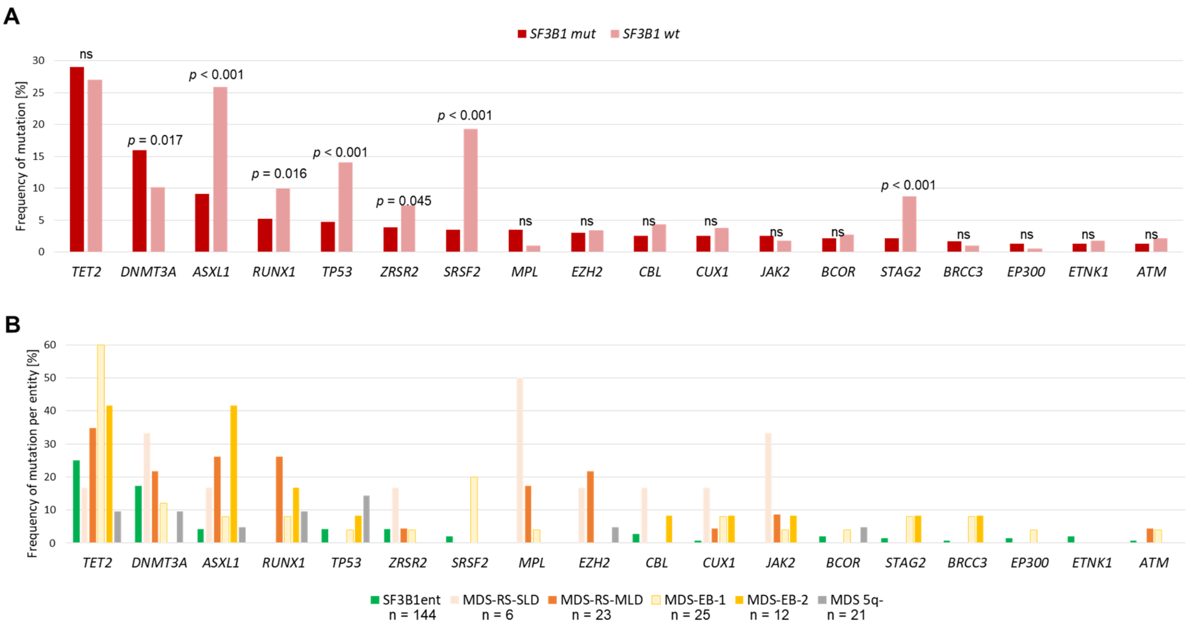

Supplementary Figure S7: Additional gene mutations in *SF3B1* mutated patients occurring in at least 3 samples. (A) Frequency of gene mutations within all *SF3B1* mutated patients (n = 231; dark red) compared to within *SF3B1* wild-type (wt) cases (n = 503; light red). (B) Frequency of gene mutations within corresponding entity.

A **B**

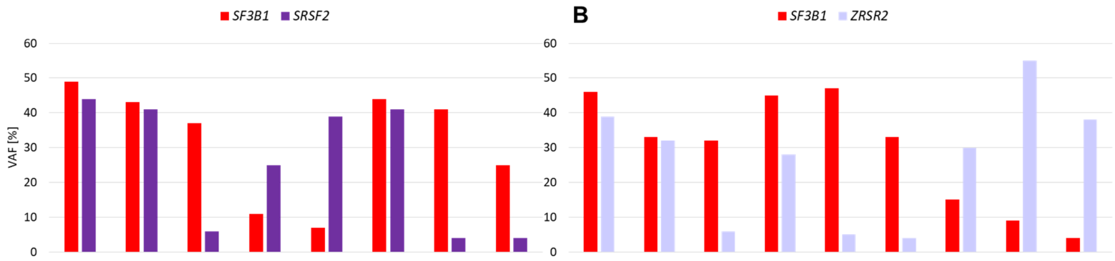

	EB-1	EB-1	EB-1	EB-1	EB-1	*SF3B1*ent	*SF3B1*ent	*SF3B1*ent	RS-MLD	EB-1	RS-SLD	*SF3B1*ent	*SF3B1*ent	*SF3B1*ent	*SF3B1*ent	*SF3B1*ent	*SF3B1*ent
SF3B1/ (SRSF2 or *ZRSR2)*	1.1	1.1	6.2	**0.4**	**0.2***	1.1	10.3	6.3	1.2	1.0	5.3	1.6	9.4	8.3	**0.5**	**0.2**	**0.1***

* AML transformation

C

	SRSF2		***ZRSR2***	
	*SF3B1*nent (n = 5)	*SF3B1*ent (n = 3)	*SF3B1*nent (n = 3)	*SF3B1*ent (n = 6)
Average VAF of *SRSF2* or *ZRSR2* (range) [%]	31 (6-44)	16 (4-41)	26 (6-39)	27 (4-55)
Average VAF of *SF3B1* (range) [%]	29 (7-49)	37 (25-44)	37 (32-46)	26 (4-47)
Ratio *SF3B1/ [SRSF2* or *ZRSR2]* (range)	1.8 (0.2-6.2)	5.9 (1.1-10.3)	2.5 (1.0-5.3)	3.3 (0.1-9.4)
Ratio *SF3B1/ (SRSF2* or *ZRSR2)* < 1.0	**n = 2**			**n = 3**

Supplementary Figure S8: *SRSF2* and *ZRSR2* mutations in *SF3B1* mutated patients. (A and B) Variant allelic frequencies (VAFs) and ratios of *SF3B1* and *SRSF2* (A) or *ZRSR2* (B) in 8 and 9 patients, respectively. (C) Summary of *SRSF2* and *ZRSR2* mutations in *SF3B1* mutated cases comparing the proposed *SF3B1* entity (*SF3B1*ent) vs. non-*SF3B1*ent (*SF3B1*nent).

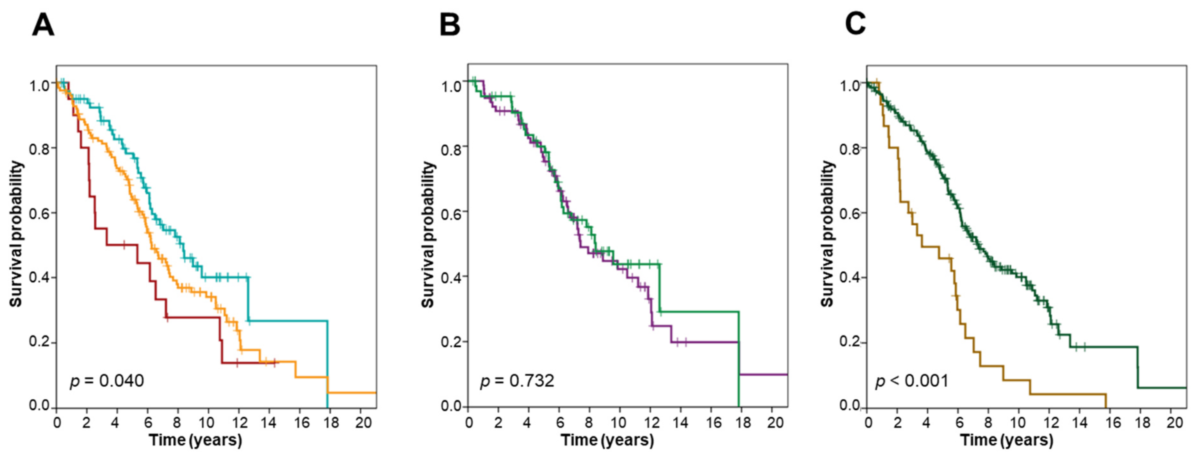

Supplementary Figure S9: Overall survival (OS) of *SF3B1* mutated MDS. (A) OS of *SF3B1*mut according to number of mutations (one mutation/ isolated *SF3B1*: n = 83, blue; 2 or 3 mutations: n = 128, orange; more than 3 mutations: n = 20, red; $p = 0.040$). (B) OS within *SF3B1*ent MDS according to isolated *SF3B1* mutation (n = 67, green) vs. *SF3B1* mutation associated with additional somatic mutations (*SF3B1* plus additional mutations: n = 77, purple) ($p = 0.732$). (C) OS of *SF3B1* mutated MDS showing either del(5q) or *RUNX1* mutations (n = 31, brown) vs. others (n = 200, dark green) ($p < 0.001$).

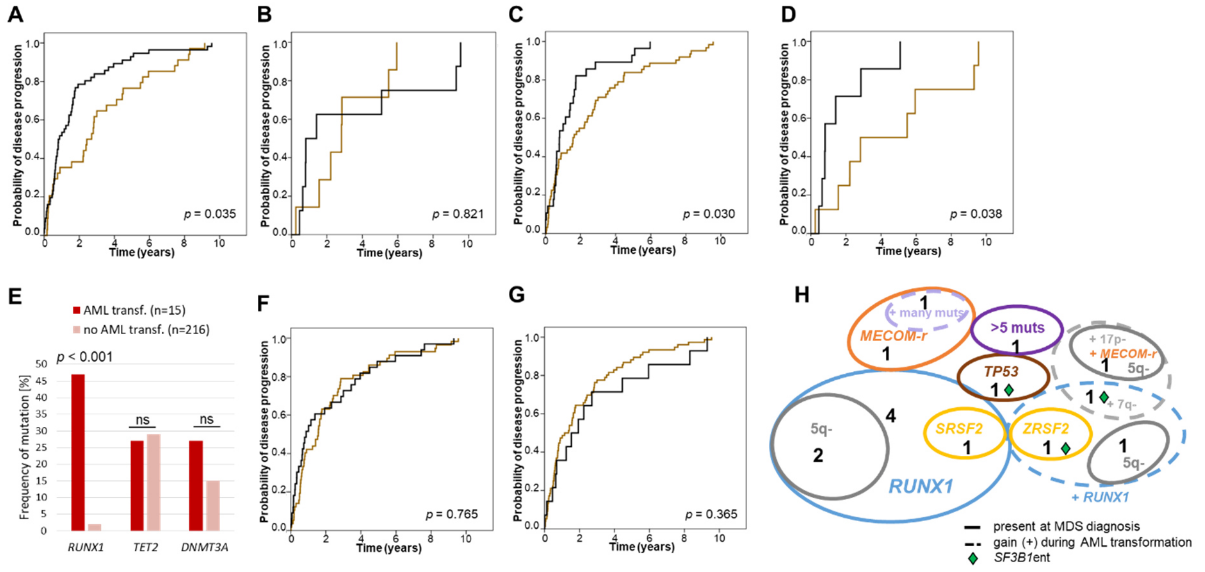

Supplementary Figure S10: Probabilities of disease progression and genetics of AML transforming *SF3B1* mutated patients. (A) Cumulative incidence of AML transformation of MDS patients having ≤ 2 mutations (n = 34; brown) vs. >2 mutations (n = 56; black). (B) Cumulative incidence of AML transformation of *SF3B1*^{mut} patients having ≤ 2 mutations (n = 7; brown) vs. >2 mutations (n = 8; black). (C) Cumulative incidence of AML transformation of *RUNX1* mutated MDS patients (n = 62; black) vs. wild-type (n = 28; brown). (D) Cumulative incidence of AML transformation of *SF3B1*^{mut} MDS patients comparing *RUNX1* mutated (n = 7; black) vs. wild-type (n = 8; brown). (E) Frequency of *RUNX1*, *TET2* and *DNMT3A* mutations in *SF3B1*^{mut} patients progressing to AML vs. patients without AML transformation (transf.); ns: not significant. (F) Cumulative incidence of AML transformation of *TET2* mutated MDS patients (n = 33; black) vs. wild-type (n = 57; brown). (G) Cumulative incidence of AML transformation of *DNMT3A* mutated MDS patients (n = 14; black) vs. wild-type (n = 76; brown). (H) Genetic landscape of 15 *SF3B1*^{mut} patients during AML transformation; r: rearrangement; mut: mutation.

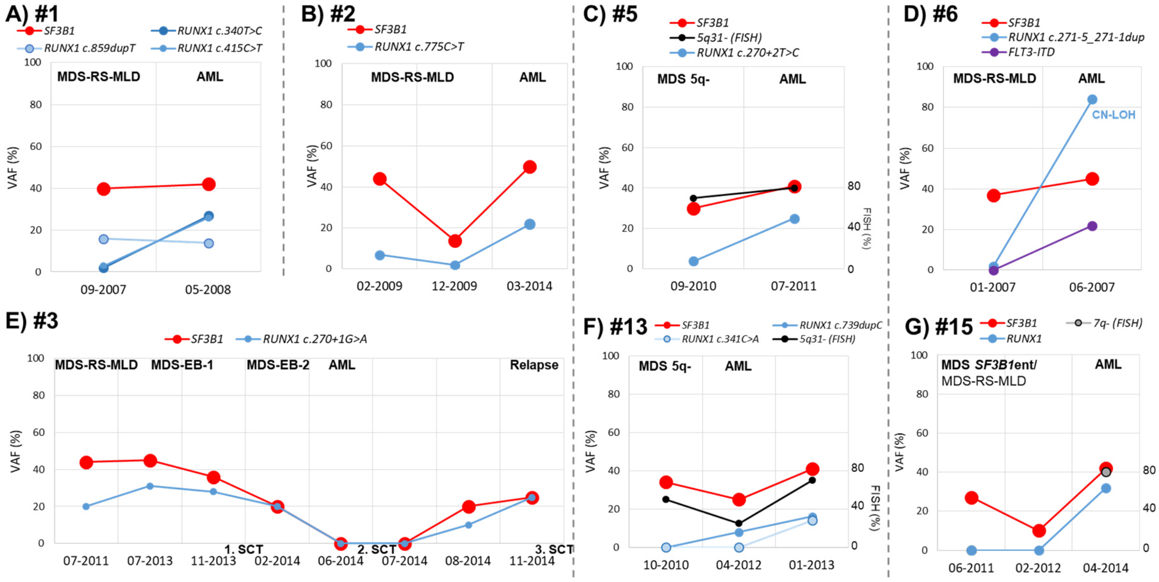

Supplementary Figure S11: Molecular genetics during disease progression of MDS patients with mutated *SF3B1*. Genetics for patient #1 (A), #2 (B), # 5 (C), #6 (D), #3 (E), #13 (F) and #15 (G) of Figure 5A. Red: *SF3B1* mutation; blue: *RUNX1* mutation; VAF: variant allelic frequency; FISH: fluorescence in situ hybridization; CN-LOH: copy neutral loss of heterozygosity; SCT: stem cell transplantation.

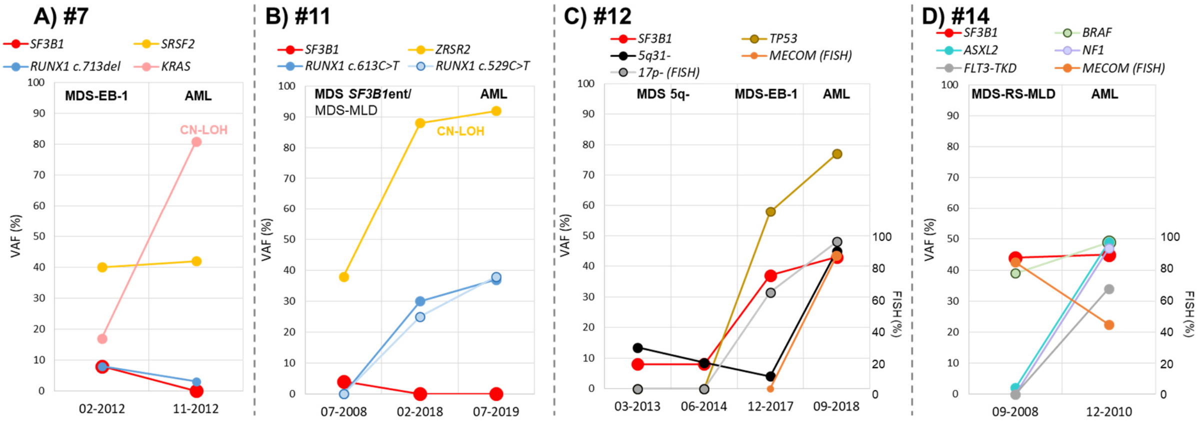

Supplementary Figure S12: Molecular genetics during disease progression of MDS patients with mutated *SF3B1*. Genetics for patient #7 (A), #11 (B), # 12 (C) and #14 (D) of Figure 5A. Red: *SF3B1* mutation; blue: *RUNX1* mutation; VAF: variant allelic frequency; FISH: fluorescence in situ hybridization; CN-LOH: copy neutral loss of heterozygosity.

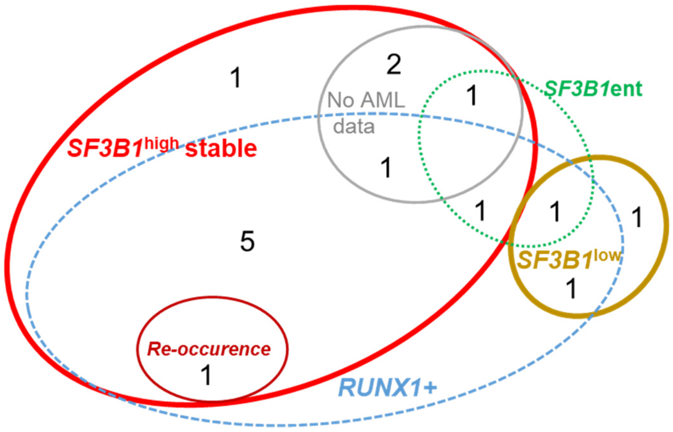

Supplementary Figure S13: Genetic landscape of *SF3B1*^{mut} patients during AML transformation focusing on *SF3B1* levels and *RUNX1* mutations. Red: high *SF3B1* VAF (n = 12); light brown: low *SF3B1* VAF (n = 3); blue: presence or gain of *RUNX1* mutations (n = 10); green: cases belonging to the proposed *SF3B1* entity (*SF3B1*ent); grey: samples without available molecular data at AML diagnosis (n = 4); VAF: variant allelic frequency.
